# At the roots of attribution of human rights to migrants

**DOI:** 10.3389/fpsyg.2022.1046616

**Published:** 2022-12-20

**Authors:** Flavia Albarello, Monica Rubini

**Affiliations:** ^1^Department of Education and Social Psychology, Sapienza University of Rome, Rome, Italy; ^2^Department of Psychology, University of Bologna, Bologna, Emilia-Romagna, Italy

**Keywords:** denial of human rights, intergroup threat, identification with the human group, in-depth exploration of identity, fraternalistic relative deprivation

## Abstract

**Introduction:**

This study (*N* = 141, *M*_age_ = 20.15) aimed at deepening knowledge on the factors that can lead young adults to deny the inalienability of human rights to migrants by examining whether, under realistic and symbolic intergroup threat (versus no-threat), the denial of human rights to migrants increases. In doing so, the role of fraternalistic relative deprivation in mediating this relation was examined. Also, two potential positive factors were considered: in-depth exploration of personal identity in the educational domain and identification with the human group. Intergroup threat was expected to enhance perceived relative deprivation, thus reducing the attribution of human rights to migrants. Such relation was expected to be mediated by those factors expressing complex views of self and others (in-depth exploration of identity in the educational domain and identification with the human group).

**Method:**

Realistic and symbolic threat were experimentally manipulated through a written scenario. In the no-threat condition, no scenario was presented.

**Results:**

Showed significant effects of intergroup threat on the attribution of human rights to migrants, on perceived fraternalistic relative deprivation, on in-depth exploration of identity in the educational domain and identification with the human group. More specifically, intergroup realistic threat, but not symbolic threat, reduced the attribution of human rights to migrants and identification with the human group. Symbolic threat, but not realistic threat, increased the perception of fraternalistic relative deprivation, whereas both realistic and symbolic threat reduced in-depth exploration of identity in the educational domain, and identification with the human group. As shown by the sequential mediation analysis, and as expected, the effect of intergroup threat in reducing attribution of human rights to migrants was mediated by in-depth exploration of identity in the educational domain, identification with the human group, and fraternalistic relative deprivation. Implications of findings concerning the processes underlying identification with the human group and its beneficial effects in terms of humanization of a stigmatized outgroup were highlighted by stressing the intertwined nature of personal identity and social identity processes. The importance of complex views of self and others in helping to create inclusive generations of adults was also highlighted.

## Introduction

Migrants represent a crucial issue for governments and society (International Organization for Migration, 2022). Wars, economic crises, and political or ethnical persecutions continuously lead people to move outside their country of origin, but recently we have witnessed a worldwide call to establish barriers against “foreigners” and defend our own nations against migrants (Annan, 2006). Not only are migrants targets of prejudice, but they are also denied membership in the human group, that is, they are dehumanized and depicted as less deserving human beings (Haslam, 2006; Albarello and Rubini, 2008, 2012b, 2015; Rubini et al., 2017; Albarello, 2020). Along this line, the acknowledgment of human deservingness and membership of migrants to the human community (Opotow, 1990) in terms of the inalienability of human rights (Albarello and Rubini, 2012a) is a core issue deserving attention given that alienation of human rights is conceived as an explicit form of dehumanization that implies withdrawal of human deservingness to others (Albarello et al., 2018a) in a conscious, propositional, and deliberative way (*cf.* Gawronski and Bodenhausen, 2006). In light of this contention, our contribution aimed to understand what factors can promote the attribution of humans rights to stigmatized social groups among young people – as the next generation of adults – by employing a cross-fertilizing approach that considers the intertwined nature of personal and social identity processes in explaining relations with others (*cf.* Albarello et al., 2021, in press-a; Crocetti et al., 2018, 2021, 2022).

Specifically, both detrimental factors that can lead to the denial of human rights to migrants and beneficial factors that can promote the humanization of such outgroupers were considered (e.g., Albarello et al., 2020; Bobba et al., 2022). First, the role of a well-known antecedent of prejudice – that is, perceived *intergroup threat* (Stephan and Stephan, 2000) – was examined. The effects of both realistic intergroup threat (threats to the ingroup’s existence, economic and political power, or physical or material well-being) and symbolic intergroup threat (threats to the values and worldviews of the ingroup) were analyzed. Moreover, looking at political discourse that often calls into play perceptions of relative deprivation (Gurr, 1970) in natives in order to mobilize people against migrants, the role of perceived *fraternalistic (or group) relative deprivation* – “the feeling that one’s group is unfairly deprived of desirable goods in comparison with relevant outgroups” (Meuleman et al., 2020, p. 593; see also Moscatelli et al., 2014) – was examined.

Not only detrimental factors, but also beneficial ones were considered. We looked at the intersection between personal and social identity processes in explaining intergroup attitudes (*cf.* Albarello et al., 2018b, 2020, 2021, in press-a, in press-b). In particular, in the current contribution, we considered *in-depth exploration of identity in the educational domain* (Crocetti et al., 2008), that is, a cognitive and motivational process at the roots of personal identity formation in the educational domain (which is the context wherein young people spend most of their time, develop their relations with others and their future commitments and identities; Albarello et al., 2018b; Crocetti et al., 2022). In-depth exploration represents the extent to which adolescents think actively about the commitments they have enacted, reflect on their choices, search for additional information about their commitments, and talk with others about them” (Crocetti et al., 2010, p. 173). Thus, through in-depth exploration, individuals actively seek and process information to make accurate and thoughtful decisions. In this vein, thinking about educational identity choices can go hand in hand with thorough information processing that can be extended to the social relational domain (*cf.* Bobba et al., 2022), for instance, to inclusive views of self and others as members of the common superinclusive group of human beings (Albarello et al., 2020): When an individual thoroughly reflects on his/her educational identity, multiple potential social categorizations applying to one’s self might become salient, thus activating cognitive complexity (Crisp and Turner, 2011; Prati et al., 2016). Such cognitive complexity can lead to reduced use of simple social categorization of others in terms of dichotomous ingroup versus outgroup categorization and more abstract, cognitively complex self-representations such as identification with the human group. This, in turn, can reduce prejudice in the heinous terms of denying humanness to others.

Given these premises, an experimental study was conducted to examine whether the activation of realistic and symbolic intergroup threat would lead to a reduced attribution of human rights to migrants. In doing so, the study considered potential detrimental (perceived fraternalistic relative deprivation) and beneficial (in-depth exploration of identity in the educational domain; identification with the human group) factors that could mediate such relation.

### Detrimental factors leading to the denial of human rights to migrants

Social psychological literature has long paid attention to antecedents of prejudice and discrimination against stigmatized social groups (Dovidio et al., 2004; Bodenhausen et al., 2009). Even though explaining attitudes towards (im) migrants and the threat caused by immigration has been a major topic of study in recent years (Allport, 1954; Vezzali et al., 2012), less attention has been dedicated to ethnic threat or even fraternalistic relative deprivation (Meuleman et al., 2020).

### Intergroup threat

The idea that real competition over scarce resources can result in ethnic conflict and negative attitudes towards outgroups has been highlighted by Sherif and Sherif’s (1964) group conflict theory. In line with this theorization, several studies (e.g., Esses et al., 2001) confirmed the relation between realistic threat and anti-immigrant attitudes, which are more widespread in adverse economic contexts (e.g., Quillan, 1995). More recently, intergroup threat has been highlighted as a factor that can explain intergroup prejudice against outgroups (for a review, see Rios et al., 2019). Meta-analytical evidence has been collected on the relation between various typologies of threat and (prejudicial) attitudes towards outgroupers (Riek et al., 2006). Stephan and Stephan’s (2000) intergroup threat theory formalized this idea that different types of threat work as antecedents of prejudice by highlighting how attitudes towards outgroups, especially ethnic groups, are affected by the perception that “migrants steal our jobs” or “overwhelm our cultural or national values” (*cf.* Esses, 2021). For instance, the criminalization of migrants, emphasizing delinquent or terrorist acts, or the risks deriving from a cultural invasion of migrants have been used by political parties as a strategy to increase support for policies against immigration in Italy (Mazzara et al., 2020), as well as in other European countries. For instance, Pereira et al. (2010), using data from a European survey, showed that the relation between prejudice and opposition to immigration was mediated by realistic threat. Using representative samples in two countries with different traditions of immigration (i.e., Switzerland and Portugal), they also highlighted that symbolic threat perception mediated the link between prejudice and opposition to the naturalization of migrants. Importantly, no study has examined whether intergroup threat enhances the tendency to deny human rights to migrants.

### Fraternalistic relative deprivation

As stated in the relative deprivation theory (Runciman, 1966), two forms of relative deprivation can be experienced depending on the reference chosen as a comparison: if egoistic relative deprivation refers to the “outcomes of intra- or interindividual comparisons” (Walker and Mann, 1987, p. 275), *fraternalistic* relative deprivation refers to the perception of (unfair) discrepancies between one’s ingroup (e.g., the national ingroup; favorite soccer team, etc.) and some other reference group (Runciman, 1966; Walker and Pettigrew, 1984; Meuleman et al., 2020). That is, fraternalistic relative deprivation might emerge as a result of comparative judgements if individuals perceive that their group is losing the status it deserves in comparison to relevant outgroups. Such perceptions can thus be unrelated to objective outcomes (Stouffer et al., 1949; Ellemers and Bos, 1998), and prejudice might derive from comparing the situation of one’s ingroup and a certain standard, that is, what people think their ingroup “should obtain.” As a consequence, fraternalistic relative deprivation has been highlighted as a factor augmenting intergroup prejudice and discrimination (Moscatelli et al., 2014), since it is possible that, independently of the objective wealth of the groups at hand, individuals perceive that they are deprived of resources that should be given to natives rather than to immigrants (Meuleman et al., 2020).

Also, the political discourse often deals with such an alleged and increasing deprivation of rights/resources of the majority group of natives (Leviston et al., 2020). In this respect, Meuleman et al. (2020) recently underlined the relation between fraternalistic relative deprivation and the perception of ethnic threat across 20 European countries. Their correlational study found a considerable association between fraternalistic relative deprivation and ethnic threat at the individual and the country levels. Nonetheless, no evidence is available on whether manipulation of intergroup threat that is thought to be caused by migrants leads people to feel fraternalistic relative deprivation nor on whether such a perception might, in turn, affect the extent to which individuals attribute inalienability of human rights to migrants (Albarello and Rubini, 2012a; Albarello et al., 2018a).

### Beneficial factors leading to the acknowledgment of human rights to migrants

Recently, attention has also been devoted to the reduction of the aggravated form of prejudice rooted in the denial of full humanness to others, that is, dehumanization (Bar-Tal, 2000; Leyens et al., 2000; Haslam, 2006; Albarello and Rubini, 2008, 2012b). For instance, Albarello and Rubini (2012a) showed that providing individuals with a definition of a Black immigrant target based on multiple belongingness (e.g., a black Christian male young person, born in Italy, having immigrant parents) reduced implicit (i.e., attribution of uniquely human secondary emotions; Leyens et al., 2000) and explicit forms (i.e., denial of human rights) of dehumanization of the Black target. Albarello and Rubini (2012a) showed that the optimal condition for reducing dehumanization towards a Black target was when human identity (*cf.* Turner et al., 1987) was made salient, and the Black target was defined with multiple categorization. Similar approaches considered human identity in different guises as a factor that can modify intergroup outcomes (e.g., Wohl and Branscombe, 2005).

A minority of studies highlighted the negative effects of activation of shared humanness. For instance, Morton and Postmes (2011) showed that perceiving shared humanness with others increased moral defense of the perpetrated harm (for similar evidence, see also Wohl and Branscombe, 2005). Focusing on the relations between victims and perpetrators, Greenaway and Louis (2010) highlighted that a benevolent representation of humanity enabled perpetrators to legitimise harm-doing to the victims yet preserving negative attitudes towards them.

On the contrary, many other contributions underlined the positive effects of acknowledgement of shared humanness. Among these, *identification with humanity* was considered as the psychological bond between the individual and the human group (Albarello et al., 2020, 2021, in press-a). Identification with all humanity is conceived as a stable individual characteristic that can be easily assessed with scales (McFarland et al., 2013; Hamer et al., 2019). Importantly, identification with all humanity has positive effects on a wide range of attitudinal and behavioral outcomes such as less prejudice, greater concern for human rights and global crises (McFarland et al., 2019). It is also associated with human rights orientation (Hamer et al., 2018) and opposition to torture (McFarland et al., 2013), etc. To the aim of highlighting its predictors, Hamer et al. (2019) conducted two cross-sectional studies intending to explain individual differences in the extent to which people identified with the superinclusive human group. They showed that openness to experience, empathy, and values such as universalism-tolerance directly enhanced identification with all humanity. In contrast, right-wing authoritarianism, social dominance orientation, and ethnocentrism partially mediated the effects of these factors on identification with humanity. Sparkman and Eidelman (2018) also showed that contact with cultural elements predicted identification with the human group, which mediated the effect of contact with cultural elements on ethnic prejudice.

If the aforementioned contributions relied on the assumption of dispositional stability of human identification (e.g., Hamer et al., 2019), other studies conceived identification with the human group as a psychological property of individuals that can be situationally activated or made salient (Albarello et al., 2018a) and vary across time depending on specific factors that might enhance or reduce it (Albarello et al., 2020, 2021). In this respect, Albarello et al. (2020) argued that identification with the human group tapped social inclusivity at a cognitive level by expressing the extent to which the individuals acknowledge their own and others’ shared membership to the human group and feel embedded within it, irrespectively of the variety of social groups encompassed in it.

For instance, recent evidence (Albarello et al., 2020) showed that prejudicial attitudes towards a stigmatized outgroup affected later identification with the human group and that the extent to which individuals identified with the human group was associated with adolescents’ social well-being (Albarello et al., 2021). That is, the more individuals identified with the human group, the more they felt integrated and able to play an active role within the communities and society they were embedded in (Keyes, 2005). Relatedly, identification with the human group should also affect the extent to which people perceive that the ingroup is relatively deprived and does not obtain what it deserves.

Most interestingly, recent studies highlighted that processes underlying both personal and social identity can jointly affect the view that individuals develop about others. Albarello et al. (2018b) showed that the processes involved in personal identity formation (i.e., commitment, in-depth-exploration of identity, reconsideration of commitment; Crocetti et al., 2008) in the educational and the relational domain (i.e., identification with classmates and friends) were deeply intertwined over time with social identity. This evidence suggests that it is worthwhile to explore their interconnections, if we aim to thoroughly unravel how individuals develop their views of themselves and others.

In particular, in the current contribution, we considered *in-depth exploration of identity* in the educational domain (Crocetti et al., 2008) – a process at the roots of personal identity formation in the educational domain, a core developmental context (Eccles and Roeser, 2011; Sani and Bennett, 2011; Crocetti, 2017). In-depth exploration of identity in the educational domain refers to the active process of thinking on current commitments, looking for additional information, and talking with others about them, in order to make accurate and thoughtful decisions. For instance, it is known that in-depth exploration of identity in the educational domain is positively associated with openness to experience (Crocetti et al., 2010; Hatano et al., 2016), a factor linked to low prejudice (e.g., Flynn, 2005). Moreover, in-depth exploration of identity in the educational domain is also conceived as a core feature (Zimmermann et al., 2012; Crocetti et al., 2013) of the information-oriented identity style (Berzonsky, 1989), whereby individuals actively seek and process relevant information to make accurate and thoughtful decisions (Berzonsky, 1989) in contrast to superficial information processing. Importantly, this style was found to be positively associated with pro-diversity and pro-equality values (Erentaitė et al., 2019) and civic engagement (Crocetti et al., 2014), and negatively associated with forms of closure to experiences or others, such as the need for cognitive closure (Crocetti et al., 2009) and different forms of prejudice (i.e., racism and xenophobia; Soenens et al., 2005). It has been shown that adolescents who adopt such an information-oriented identity style also have a high degree of cognitive complexity and employ a vigilant and systematic processing style in decisional situations (Berzonsky and Ferrari, 1996); thus, it can be assumed that they are also less susceptible to use social cognitive simplifications that lead to social prejudice (Allport, 1954).

In this vein, in-depth exploration of identity in the educational domain might go hand in hand with the dismissal of cognitive simplifications of social reality (e.g., ingroup bias, social stereotypes, etc.) and might be connected to more inclusive views of others, for instance, identification with the common superinclusive human ingroup (Albarello et al., 2020; *cf.* Gaertner and Dovidio, 2000). In other words, resembling the effect of openness to experience on identification with all humanity highlighted by Hamer et al. (2019), it could be possible that high in-depth exploration of identity in the educational domain leads to high identification with the human group.

Moreover, situational threats (e.g., enhanced salience of intergroup threat due to migrants) might affect the extent to which individuals proceed in exploration processes, for instance enhancing the individuals’ need for cognitive closure (Mula et al., 2022; see also Albarello et al., 2022), leading – in turn – to enhanced negative attitudes towards migrants. Thus, it could be expected that the salience of intergroup threat (realistic or symbolic) reduces individuals’ in-depth exploration of identity in the educational domain to maintain certain, secure knowledge (Webster and Kruglanski, 1994) on individuals’ views about oneself and own educational choices.

## The current study

Given these premises, an experimental study was conducted on a sample of first-year university students to examine whether realistic and symbolic intergroup threat (versus no-threat) lead to a reduced attribution of the inalienability of human rights to migrants. Realistic and symbolic threat were manipulated through previously employed scenarios (Albarello et al., 2017, 2019; Albarello and Rubini, 2018). In order to deepen knowledge of the psychological processes that lead individuals to deny human rights to migrants when threats (e.g., realistic and symbolic threat) are activated, the study also considered detrimental (fraternalistic relative deprivation) and beneficial (in-depth exploration of identity in the educational domain; identification with the human group) factors that could mediate such relation.

Specifically, given that threat has been shown to restrict intergroup boundaries and increase prejudicial attitudes towards migrants (e.g., Pereira et al., 2010; Ho et al., 2013), it was expected that realistic and symbolic threat would lead individuals to refrain from attributing human rights to migrants in contrast to the absence of threat (*hypothesis 1*). In this respect, considering that recent correlational evidence suggested that ethnic threat and natives’ relative deprivation compared to migrants were associated (Meuleman et al., 2020), we expected that under intergroup threat, the perception of own national ingroup’s relative deprivation would be enhanced (*hypothesis 2*). Based on this, we also expected that the effect of threat on the attribution of inalienability of human rights to migrants would be mediated by natives’ increased feeling of being relatively deprived (*hypothesis 3*).

Since intergroup threat has various effects on the mind, brain and behavior of social perceivers (*cf.* Chang et al., 2016) related to group-protection and self-protection motives (e.g., high tendency to over-exclude potentially dangerous outgroupers; Krosch and Amodio, 2014; Albarello et al., 2019), it could also hinder the exploration of identity alternatives in the educational domain as a self-protective reaction (*hypothesis 4a*) and reduce the extent to which people identify with the common superinclusive ingroup that includes also migrants (*hypothesis 4b*).

Moreover, since in-depth exploration of identity in the educational domain represents a core feature of the information-oriented identity style (Berzonsky, 1989; Crocetti et al., 2013) leading to the dismissal of cognitive simplifications of social reality, we expected that it would affect the extent to which individuals identify with the human group, thus mediating – in this sequence – the relation between threat and attribution of human rights (*hypothesis 5*).

Given that identification with the human group represents the extent to which a person acknowledges his/her own and others’ membership in the most inclusive ingroup (Turner et al., 1987; Albarello et al., 2020), it could, in turn, lessen the perception of fraternalistic relative deprivation. That is, we expected the effect of threat on the attribution of inalienability of human rights to migrants to be sequentially mediated by in-depth exploration of identity in the educational domain, identification with the human group, and perceived fraternalistic relative deprivation (*hypothesis 6*).

## Materials and methods

### Participants and procedure

One hundred and forty-one first-year Italian university students (*M*_age_ = 20.15, *SD* = 0.89, females: 75.2%) participated in the study voluntarily. A convenience sample was recruited during university lectures on social psychology of communication during the second spread of the Coronavirus (COVID-19) disease in the spring of 2021. Post-hoc power analysis with G*Power entering the sampled participants (*N* = 141), a medium size effect’s size (0.30), and the three group conditions (no-threat, realistic threat, symbolic threat) revealed that the reached power (1 - β error probability) was equal to 0.75. Participants with non-Italian nationality were excluded from the sample. The majority of participants were full-time university students (*n* = 130; 92.2%), and only a minority also had a job (*n* = 11; 7.8%). Participants came from 11 out of the 20 Italian regions and mainly from central regions of Italy (*n*_center_ = 82; 58.2%), followed by southern regions (*n*_south_ = 57; 40.4%) and northern regions (*n*_north_ = 2; 1.4%). The most represented region was Lazio (center of Italy, *n* = 70), followed by Campania (south of Italy, *n* = 22). The distribution of participants and the descriptives referred to the study’s variables depending on the region of origin of respondents are reported in Table 1.

**Table 1:** Descriptive statistics for each Italian region.

Italian region	*N*	% of respondents from each Italian region	*n* males	*n* females	Age	In-Depth exploration of identity in the educational domain	Identification with the human group	Fraternalistic relative deprivation	Inalienability of human rights to migrants
					*M(SD)*	*M(SD)*	*M(SD)*	*M(SD)*	*M(SD)*
Liguria	2	1,4	0	2	19.50 (0.71)	3.80 (0.56)	3.50 (1.06)	2.25 (0.35)	4.67 (0.47)
Tuscany	4	2,8	1	3	20.75 (1.50)	3.55 (0.44)	3.38 (1.61)	2.25 (0.87)	4.92 (0.17)
Umbria	8	5,7	1	7	20.38 (0.52)	4.03 (0.55)	4.19 (0.69)	1.44 (0.58)	5.00 (0.00)
Abruzzo	7	5,0	3	4	20.00 (0.58)	3.57 (0.68)	3.82 (0.70)	2.04 (0.78)	4.55 (0.58)
Lazio	70	49,6	18	52	20.11 (0.92)	3.58 (0.54)	3.89 (0.78)	1.84 (0.78)	4.80 (0.50)
Basilicata	4	2,8	0	4	20.00 (0.00)	3.85 (0.44)	3.63 (0.47)	1.69 (0.85)	4.95 (0.08)
Campania	22	15,6	5	17	20.14 (0.77)	3.76 (0.60)	3.99 (0.58)	1.81 (0.75)	4.84 (0.40)
Puglia	9	6,4	3	6	20.56 (1.33)	3.87 (0.39)	4.11 (0.61)	1.64 (0.70)	4.96 (0.11)
Calabria	5	3,5	1	4	20.00 (0.00)	3.20 (1.26)	3.45 (0.76)	2.95 (1.15)	4.30 (1.04)
Sicilia	6	4,3	2	4	20.00 (0.00)	3.63 (0.53)	3.83 (0.83)	2.29 (1.05)	4.83 (0.41)
Sardegna	4	2,8	1	3	20.00 (0.00)	3.50 (0.50)	3.44 (0.72)	1.88 (0.43)	4.54 (0.81)
Total	164	100	35	106	20.14 (0.86)	3.88 (0.76)	1.88 (0.80)	4.80 (0.48)	3.65 (0.59)

The study’s variables of interest did not vary depending on the region of origin of respondents, as shown by one-way ANOVAs (region), *F_s_* (10, 130) ≤ 1.67, *p_s_* ≤ 0.095, *η*^2^ = 0.114.

Participants were asked to fill-in an anonymous online questionnaire on Qualtrics aimed to assess issues related to the self and relations with others. Once participants gave their informed consent, they were exposed to the threat manipulation and were randomly assigned to one of three conditions: no-threat, realistic threat, symbolic threat. Both realistic and symbolic threat were manipulated through a written scenario employed in previous research (Albarello et al., 2017, 2019; Albarello and Rubini, 2018). The realistic threat scenario referred to the threat posed by migrants in terms of unemployment and costs of health and social welfare. The symbolic threat scenario referred to the cultural differences between migrants and natives. In the no-threat condition, no-scenario was presented.

Specifically, the realistic threat scenario read as follows: “Recent research by the national statistical institute showed that during the past year unemployment increased for Italians (+3%) and 176.000 Italians lost their jobs. Conversely, migrants’ employment level increased (+200.000). Moreover, migration led to increased costs for public health, education, and welfare policies aimed to promote migrants’ integration.” The symbolic threat scenario read as follows: “Recent research by the national statistical institute showed strong cultural differences between Italians and migrants. Migrants have different habits, traditions, ideologies, and moral values when compared with those of Italians. Migrants are also radically different in terms of their lifestyles, the ways in which they behave at work and also at home, for instance, in terms of the children’s educational policies they endorse.”

Subsequently, participants filled out a questionnaire aimed to collect the research measures of interest (as listed below) and participants’ demographics. The last part of the questionnaire thanked and carefully debriefed participants. In order to obtain the course credit, participants were then redirected to a different section of the questionnaire on Qualtrics wherein they could provide their names and e-mails.

## Measures

### In-depth exploration of identity in the educational domain

In-depth exploration of identity choices in the educational domain was measured with the in-depth exploration of identity in the educational domain subscale of the Utrecht-Management of Identity Commitments Scale (U-MICS, Crocetti et al., 2008; Italian validation by Crocetti et al., 2010). The instrument consists of five items (α = 0.70) scored on a 5-point Likert-type rating scale, ranging from 1 (*completely false*) to 5 (*completely true*). Sample items include: “I think a lot about my education,” “I am interested in deeply understanding the value of my formation.”

### Identification with the human group

To assess identification with the human group, the four-item identification with the human group scale (Albarello and Rubini, 2012a) was employed (α = 0.78). This scale was originally developed in Italian. Sample items are: “I identify with all human beings,” “I am like all human beings, irrespectively of ethnic, political, religious, social or ideological differences.” Participants rated the items on a 5-point Likert-type rating scale from 1 (*completely false*) to 5 (*completely true*).

### Fraternalistic relative deprivation

Fraternalistic relative deprivation was assessed with a four-item scale (α = 0.92) inspired by the work by Ellemers and Bos (1998). Sample items are: “In general, I think that the situation of Italians has become more critical as a consequence of the increased number of migrants in the territory” and “In general, I think that the outcomes of Italians are worse than those they are entitled to as a consequence of the increased number of migrants in the territory.” Participants rated the items on a 5-point Likert-type rating scale from 1 (*not at all*) to 5 (*very much*).

### Inalienability of human rights

The attribution of the inalienability of human rights to the outgroup of migrants was assessed with six items taken from the scale developed by Albarello and Rubini (2012a). The six items (e.g., “All human beings are born free and equal in dignity and rights”; “Everyone is entitled to rights and freedoms, without distinction of any kind as regards to race, colour, sex, language, religion, political or other opinions”; α = 0.95) were taken in order to reduce the length of the questionnaire. Alpha of the scale was comparable to that of the original 10-items version (α = 0.98; *cf.* Albarello and Rubini, 2012a). Participants rated the items on a 5-point Likert-type rating scale from 1 (*not at all*) to 5 (*very much*).

### Manipulation checks and demographics

As in previous research (Albarello et al., 2019), participants rated on 5-point Liker-type scales (1 = *not at all,* 5 = *very much*) the extent to which they experienced “fear” and “concern” (α = 0.85) reading the information provided at the beginning of the research session (i.e., description of the groups and information on migrants). Participants’ demographics (i.e., age, gender, occupation, place of residence) were also assessed (see Table 1).

## Results

### Preliminary analyses

One-way (threat: no-threat, realistic threat, symbolic threat) Analyses of Variance (ANOVAs) were conducted on all the dependent variables. Importantly, the results showed that the mean score of experienced threat due to threat manipulation significantly differed among the three threat conditions (*M*_no-threat_ = 1.99, *SD* = 1.09; *M*_realistic threat_ = 3.03, *SD* = 1.15; *M*_symbolic threat_ = 3.29, *SD* = 0.62), *F* (2, 138) = 25.49, *p* < 0.001, η^2^ = 0.270. Participants in both the realistic threat and symbolic threat conditions felt more worried than in the no-threat condition (*p*_s_ < 0.001 at the Bonferroni post-hoc comparisons).

As highlighted in Table 2, intergroup threat significantly reduced the attribution of human rights to migrants (*hypothesis 1*) in comparison with the no-threat condition (*M*_no-threat_ = 4.93, *SD* = 0.22; *M*_realistic threat_ = 4.60, *SD* = 0.70; *M*_symbolic threat_ = 4.74, *SD* = 0.55), *F* (2, 138) = 5.76, *p* = 0.004, η^2^ = 0.077. As expected, participants in the realistic threat condition attributed human rights to migrants to a lower extent than in the no-threat condition (*p* = 0.004 at the Bonferroni post-hoc comparison). On the contrary, the comparison between the symbolic threat condition and the no-threat one did not reach the statistical significance (*p* = 0.127). Also no difference emerged between the realistic and the symbolic threat conditions (*p* = 0.690).

**Table 2:** In-depth exploration of identity in the educational domain, identification with the human group, fraternalistic relative deprivation, and inalienability of human rights to migrants as a function of intergroup threat.

	In-depth exploration of identity in the educational domain	Identification with the human group	Fraternalistic relative deprivation	Inalienability of human rights to migrants
No-threat	3.92_a_ (0.49)	4.07_a_ (0.74)	1.70_a_ (0.75)	4.93_a_ (0.22)
Realistic threat	3.34_b_ (0.47)	3.48_b_ (0.85)	2.05_ab_ (0.75)	4.60_b_ (0.69)
Symbolic threat	3.42_b_ (0.65)	3.86_ab_ (0.57)	2.06_b_ (0.90)	4.74_ab_ (0.55)

Standard deviations are reported in parentheses. Means with different subscripts within columns significantly differed (*p* < 0.05).

Intergroup threat also enhanced the extent to which participants felt that their national ingroup was relatively deprived *(hypothesis 2)*, if compared to the no-threat condition (*M*_no-threat_ = 1.70, *SD* = 0.72; *M*_realistic threat_ = 2.05, *SD* = 0.75; *M*_symbolic threat_ = 2.06, *SD* = 0.91), *F* (2, 138) = 3.62, *p* = 0.029, η^2^ = 0.050. Bonferroni post-hoc comparisons showed a marginally significant difference in the expected direction between the symbolic and the no-threat conditions (*p* = 0.072). In contrast, the realistic threat condition did not differ from both the no-threat (*p* = 0.109) and the symbolic threat (*p* = 1.00) conditions.

A significant main effect of intergroup threat in reducing in-depth exploration of identity in the educational domain in contrast to the no-threat condition (*M*_no-threat_ = 3.92, *SD* = 0.49; *M*_realistic threat_ = 3.34, *SD* = 0.45; *M*_symbolic threat_ = 3.42, *SD* = 0.65) emerged, *F* (2, 138) = 28.60, *p* < 0.001, η^2^ = 0.212. Importantly, as expected (*hypothesis 4a*), participants in both the realistic and the symbolic threat conditions reported lower in-depth exploration of identity in the educational domain (*p*_s_ < 0.001 at the post-hoc Bonferroni comparison) than those in the no-threat one.

Intergroup threat also reduced identification with the human group (*hypothesis 4b*), in comparison to the no-threat condition (*M*_no-threat_ = 4.07, *SD* = 0.74; *M*_realistic threat_ = 3.48, *SD* = 0.85; *M*_symbolic threat_ = 3.86, *SD* = 0.57), *F* (2, 138) = 7.43, *p* = 0.001, η^2^ = 0.097. Bonferroni post-hoc comparisons highlighted that the realistic threat condition significantly differed from the no-threat one (*p* = 0.001). The comparison between the realistic and the symbolic threat conditions was marginally significant (*p* = 0.086). In contrast to the expectations, the symbolic threat condition did not differ from the no-threat one (*p* = 0.405).

Importantly, as shown by Pearson’s correlation analysis (see Table 3), high levels of in-depth exploration of identity in the educational domain corresponded to high levels of identification with the human group (*r* = 0.298, *p* < 0.001) and attribution of human rights to migrants (*r* = 0.184, *p* = 0.029). Participants with high identification with the human group reported lower fraternalistic relative deprivation (*r* = −0.245, *p* < 0.001), and rated higher inalienability of human rights to migrants (*r* = 0.279, *p* = 0.001). Those who reported higher fraternalistic relative deprivation attributed lower inalienability of human rights to migrants (*r* = −0.333, *p* < 0.001). Overall, such associations allowed us to test the specific sequential mediational paths that had been hypothesized.

**Table 3:** Pearson’s correlations among study’s variables (*N* = 141).

	In-depth exploration of identity in the educational domain	Identification with the human group	Fraternalistic relative deprivation	Inalienability of Human rights to migrants
In-depth exploration	−			
Identification with the human group	0.289^***^	−		
Fraternalistic relative deprivation	0.005	−0.245^**^	−	
Inalienability of Human rights	0.184^*^	0.275^**^	−0.333^***^	−

^*^p < 0.05, ^**^p < 0.01, ^***^p < 0.001.

### Mediational analysis

**Figure 1:**
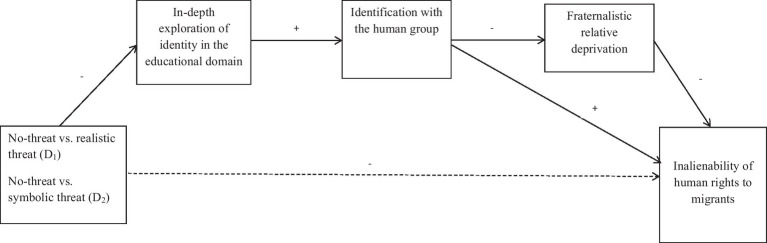
Conceptual multiple step mediation model of the effect of intergroup threat (dummy coded as D_1_ and D_2_) on inalienability of human rights to migrants through in-depth exploration of identity in the educational domain, identification with the human group, and fraternalistic relative deprivation. D_1_, no-threat (0), realistic threat (1); D2, no-threat (0), symbolic threat (1).

In order to test the expected mediational paths (*hypothesis 3, hypothesis 5, hypothesis 6*), bootstrapping sequential mediational analyses (5,000 re-samples) testing direct and indirect effects in multiple step mediations models – as prescribed by Hayes et al. (2011) – were conducted. The expected conceptual multiple step mediational model is represented in Figure 1. Using the method described by Hayes and Preacher (2014) for mediational models employing multicategorical independent variables, the independent variable was recoded as two dummy variables: D_1_ contrasted the no-threat (coded 0) and realistic threat (coded 1) conditions; D_2_ compared the no-threat (coded 0) and the symbolic threat (coded 1) conditions. D_1_ and D_2_ were entered simultaneously as predictors in the regression model. The PROCESS 3.3 macro for SPSS (model 6) was used, since it produces omnibus tests of total and direct effects indicating whether there is an effect of the independent variables on the outcome variable without specifying which dummy variable is responsible for it.

As shown in Table 4, this analysis highlighted significant relative total effects of both D_1_ and D_2_ on the attribution of inalienability of human rights to migrants, respectively, *B* = −0.32, *SE* = 0.10, and *B* = −0.19, *SE* = 0.09; *p_s_* ≥ 0.042. The omnibus test of total effect of D_1_ and D_2_ was significant, *F* (2, 138) = 5.76, *p* = 0.004, *R*^2^ = 0.08. When the mediators were included in the model, the omnibus test of direct effects of D_1_ and D_2_ turned to non-significance, *F* (2, 135) = 1.28, *p* = 0.282, *R*^2^ = 0.02, and the relative direct effects of D_1_ and D_2_ on the outcome variable became non-significant, *p*_s_ ≥ 0.113.

**Table 4:** Total, direct, and indirect effects of predictors of inalienability of human rights to migrants.

	Total effect			Direct effect			Indirect effect 95% CI through fraternalistic relative deprivation				Indirect effect 95% CI through in-depth exploration of identity in the educational domain and identification with the human group				Indirect effect 95% CI through in-depth exploration of identity in the educational domain, identification with the human group, and fraternalistic relative deprivation			
Predictor	*B*	*SE*	*p*	*B*	*SE*	*p*	*B*	*SE*	*LL*	*UL*	*B*	*SE*	*LL*	*UL*	*B*	*SE*	*LL*	*UL*
D_1_	−0.32	0.10	0.002	−0.17	0.11	0.113	−0.06	0.04	−0.16	−0.00	−0.02	0.01	−0.04	0.00	−0.01	0.01	−0.02	−0.00
D_2_	−0.19	0.09	0.042	−0.08	0.10	0.423	−0.07	0.05	−0.10	−0.00	−0.02	0.01	−0.04	0.00	−0.01	0.01	−0.02	−0.00

D1, no-threat (0), realistic threat (1); D2, no-threat (0), symbolic threat (1); B, unstandardized beta weights; SE, standard error; LL, lower limit; UL, upper limit

As for the specific indirect effects, the analysis revealed, as expected (*hypothesis 3*), that the indirect effect of threat through fraternalistic relative deprivation was significant both for D_1_ and D_2_ (respectively, *B* = −0.06, *SE* = 0.01; *B* = −0.07, *SE* = 0.05), since the 95% Confidence intervals (CI) did not include zero, respectively [−0.16, −0.00] and [−0.18, −0.00].

Contrary to expectation (*hypothesis 5*), the sequential indirect effect of threat through in-depth exploration of identity in the educational domain and, in turn, identification with the human group, was not significant, with the 95% CIs including zero both for D_1_, *B* = −0.02, *SE* = 0.01, 95% CI [−0.04, 0.00], and for D_2_, *B* = −0.01, *SE* = 0.01, 95% CI [−0.04, 0.00]. Even if not predicted, the analysis revealed a further significant sequential indirect effect of threat on the attribution of human rights to migrants through in-depth exploration of identity in the educational domain and, in turn, fraternalistic relative deprivation both for D_1_, *B* = 0.02, *SE* = 0.02, 95% CI [0.00, 0.07], and D_2_, *B* = 0.02, *SE* = 0.02, 95% CI [0.00, 0.06].

Most importantly, and as expected (*hypothesis 6*), the bootstrapping mediational analysis revealed a significant sequential indirect effect of threat on attribution of human rights to migrants through in-depth exploration of identity in the educational domain, identification with the human group, and fraternalistic relative deprivation both for D_1_, *B* = −0.01, *SE* = 0.00, 95% CI [−0.02, −0.00], and D_2_, *B* = −0.01, *SE* = 0.00, 95% CI [−0.02, −0.00].^1^

Overall, evidence of this analysis highlighted that the effects of both realistic and symbolic intergroup threat in reducing attribution of human rights to migrants were mediated by fraternalistic relative deprivation, a factor eliciting salience of intergroup distinction. Nonetheless, this path was also challenged by processes involved in individuals’ personal identity formation in terms of thoughtful exploration of identity choices and by high levels of identification with the human group, with all this entails in terms of acceptance of different outgroupers within the common human ingroup.

## Discussion

This study aimed to tackle the underexplored association between realistic and symbolic intergroup threat as antecedents of a negative form of discrimination such as dehumanization, expressed as the denial of human rights (Albarello and Rubini, 2012a) to migrants by young adults.

Intergroup threat was conceived as a factor that might increase the denial of human rights to migrants (*cf.* Albarello et al., 2022; Mula et al., 2022), leading to various adverse outcomes as the ones highlighted in this contribution. By endorsing a cross-fertilizing approach that combined insights deriving from personal and developmental psychology with a social identity approach to intergroup relations, we aimed to analyse whether the effect of intergroup threat on the denial of human rights to migrants could be mediated. To this aim, we considered fraternalistic relative deprivation (Moscatelli et al., 2014) as a detrimental factor, as well as beneficial processes related to complex views of self and others (Albarello et al., in press-a) as in-depth exploration of identity in the educational domain (Crocetti et al., 2008; Bobba et al., 2022) and identification with the human group (Albarello et al., 2020, 2021).

Results highlighted that realistic threat led to a reduced attribution of human rights to migrants, and it increased the perception that the ingroup of natives was relatively deprived. Moreover, it reduced in-depth exploration of identity in the educational domain, and led to lower identification with the human group. That is, it restricted intergroup boundaries leading to the exclusion of the outgroup of migrants from the common ingroup of human beings (Opotow, 1990) in terms of denial of human rights, as well as reducing the extent to which young adults acknowledge their own belongingness to the human group.

Symbolic threat seemed less effective in leading to these negative outcomes, since its effects in increasing the denial of human rights of migrants and reducing identification with the human group were not fully significant. Nonetheless, it reduced the extent to which young adults reported questioning their educational choices.

Most importantly, the findings highlighted the paths through which intergroup threat can lead to denial of human rights to migrants by highlighting the detrimental role of fraternalistic relative deprivation as a consequence of threat activation directly on reduced attribution of human rights. Results showed that this is only one possibility, since further indirect sequential mediational effects emerged. Findings showed that intergroup threat, both realistic and symbolic, froze in-depth exploration of identity choices leading, in turn, to increased fraternalistic relative deprivation. Moreover, threat led to the denial of human rights to migrants by reducing in-depth exploration of identity, thus leading to lower identification with the human group, which in turn affected the extent to which young adults felt that the ingroup of natives was relatively deprived, increasing denial of human rights to migrants.

Nonetheless, focusing on the no-threat condition, findings also underlined that the effect of threat could be contrasted by a beneficial path involving exploration of own personal identity choices in the educational domain (Crocetti et al., 2008), which had the positive outcome of fostering complex and more abstract (*cf.* Albarello et al., 2020, 2021) self-definitions. Consequently, the perception of relative deprivation of the ingroup of natives was reduced, hindering the dehumanization of the minority outgroup of migrants when intergroup threat was not activated.

This study showed that threat led to explicit dehumanization of migrants in terms of denial of human rights by hindering the beneficial effect of factors that can challenge the dichotomous view of “us versus them,” such as in-depth exploration of personal identity choices in the educational domain and identification with the common human group, leading to higher perception of fraternalistic relative deprivation.

Overall, this evidence suggests that the psychological processes leading to the restriction of intergroup boundaries and exclusion of outgroups from the human community (Opotow, 1990) – the most important good that can be acknowledged to others (Walzer, 1983) – is very complex and rooted in the intertwined nature of personal and social identity processes (Albarello et al., 2018b, 2021; Crocetti et al., 2022). This evidence thus stresses the importance of considering both personal and social processes to thoroughly understand the underlying processes that can lead to (de) humanization of stigmatized outgroupers. In this vein, these findings underline that developing complex views of self and others (*cf.* Albarello et al., in press-a) can help create inclusive generations of adults.

### The role of complex views of self and others in promoting attribution of human rights

By deepening knowledge of the destructive path that leads to the denial of human rights to migrants when a threatening situation is at stake, this study also highlights that beneficial processes related to personal and social identifications can challenge dehumanization of stigmatized outgroupers widening of one’s moral community (Opotow, 1990). This contribution provides the pivotal evidence that the more individuals think thoroughly about themselves and their identities, both in terms of a comprehensive identity exploration and acquisition of abstract identifications, the more they acknowledge human deservingness to outgroupers, expressed in complex, ideological terms – such as attribution of human rights – and the less they rely on ingroup categories and suffer from the negative outcome of perceived relative deprivation of their ingroup. These are thus crucial identity resources, like an “identity baggage” that young adults might develop to become less prejudiced adults.

In this respect, interventions that foster exploration of identity choices in various domains of an individual’s life experience (including the educational one, as we showed) and promote individuals’ identification with the most inclusive common ingroup – the human group – help disrupting the negative consequences of the factors that enhance salience of intergroup boundaries, such as intergroup threat and its negative consequences (e.g., arising of feelings of relative deprivation).

Importantly, this study highlights the positive association between a thorough investigation of own identity and self-identification with the human ingroup as resources against the dehumanization of migrants. Future studies should build on this pivotal evidence by developing manipulations of in-depth personal identity exploration. Moreover, measures of identity exploration in broader and less specific domains might be developed to be able to assess the mechanism through which identity exploration can work as a beneficial factor in challenging prejudice, also in its more extreme forms such as dehumanization.

Future contributions should tackle more directly the sources of such exploration processes. In this vein, in-depth exploration of identity might be associated with epistemic quests, like the one characterizing the seizing phase of individual’s need for cognitive closure (Webster and Kruglanski, 1994). Epistemic quest starts when individuals are confronted with a question to which they do not have an answer and stops when the answer is found. In this respect, two phases of this quest have been highlighted: the seizing one, in which individuals are motivated to find an answer that can provide stable knowledge in the present and the future, and the freezing phase, when they are instead motivated to defend their existing knowledge. In this vein, in-depth exploration of identity might be related to quest processes characterizing the seizing phase, thus explaining the connection with higher human identification, as the acknowledgment of self and others’ belongingness to the human group, with the commonalities and differences that this entails.

### Into the negative spiral of threats

Overall, this study showed that situational threats (e.g., intergroup threat) might challenge and stop such advanced, thorough cognitive processing of own and others’ identities. This was particularly evident in the realistic threat condition. Symbolic threat’s effects were less potent than the ones of realistic threat. This could be related to the specific events that happened when data were collected. During the last two years, several global crises have been experienced: The recent war in Ukraine, as well as the pandemic due to the COVID-19, have been perceived as global threats leading people to experience concern and uncertainty (Mula et al., 2022). Such experiences might have sensitized people and young adults to conceive them as threats to themselves and ingroups’ well-being and survival. This is in line with evidence showing that perceived threat is associated with greater intolerance and punitiveness towards outgroups (Marcus et al., 1995; Feldman and Stenner, 1997; Jackson et al., 2019), as well as higher ethnocentrism (Schaller and Neuberg, 2012). For instance, it has been also shown that concern with COVID-19 threat, by leading to higher desire for cultural tightness (Gelfand et al., 2011), also enhances prejudice towards immigrants (Mula et al., 2022). In this vein, other threats (e.g., COVID-19, ecological disasters, wars, etc.) might hinder the beneficial paths underlined in this study.

In this vein, future studies should disentangle the role of various types of threat and the role of perceptions of threatening factors/events on in-depth exploration of identity, identification with the human group, and dehumanization of outgroups. Acknowledgment of the potential boost effect from various contextual society-threatening events on prejudice and discrimination could be of utmost importance to timely set effective social policies aimed to promote harmonious intergroup relations in modern globalized societies. This would be very informative for social agendas, as well as for scholars’ ones, since it means that the study of prejudice and discrimination still needs to be updated and contextualized to actual global social issues.

## Limitations

Notwithstanding the aforementioned important implications, this study has several flaws that might be more carefully tackled in future research. It might be worthwhile to address why the effect of symbolic threat seemed less effective than the realistic threat in reducing the attribution of human rights to migrants. Moreover, studies with larger samples might be conducted. A convenience sample was employed to reach participants to whom in-depth exploration of identity in the educational domain could apply. Participants were recruited during university lectures during the COVID-19 lockdowns in Italy in the spring of 2021. This constraint made it difficult to enlarge the sample without providing monetary rewards. Moreover, the sample mainly comprised females. A more balanced sampling in terms of respondents’ gender should be obtained. Nonetheless, the findings of this study are noteworthy since they show that also females, who are usually regarded as more “communal” (e.g., Suitner and Maass, 2008) than males, react to situational threats restricting the scope of application of human rights to migrants, thus refusing to include them into their human moral community (Staub, 1989; Opotow, 1990).

Cross-cultural studies would also be needed. In particular, the actual percentage of immigrants in respondents’ place of residence might be included in research as a second-level variable that might help address the factors that lead young people to dehumanize migrants when intergroup threat is made salient. In this vein, the nature of actual contact (positive or negative) with (im) migrants should also be considered in future studies.

## Conclusion

This contribution provides pivotal evidence of the complex and intertwined nature of personal and social identity processes in leading to the attribution or denial of human deservingness to a very salient outgroup, migrants – who are still the targets of widespread prejudice, which can be risen by activation of various types of threat (e.g., intergroup symbolic or realistic threat, etc.). In this vein, this contribution paves the way for a fruitful integration of different theoretical approaches in tackling prejudice and aggravated forms of discrimination such as dehumanization. Endorsing a complex view of self and others might represent a “safety-baggage” against such prejudices by contrasting the effects of dichotomous ingroup versus outgroup categorizations, at least when threat is not activated.

## Data availability statement

The raw data supporting the conclusions of this article will be made available by the authors, without undue reservation.

## Ethics statement

The studies involving human participants were reviewed and approved by University of Bologna, Ethics Commitee of the Department of Psychology. The participants provided their written informed consent to participate in this study.

## Author contributions

FA and MR conceived of the current study and participated in its design and coordination. FA performed the statistical analyses, and wrote the manuscript. MR helped to draft the manuscript. All authors read and approved the final manuscript.

## Conflict of interest

The authors declare that the research was conducted in the absence of any commercial or financial relationships that could be construed as a potential conflict of interest.

## Publisher’s note

All claims expressed in this article are solely those of the authors and do not necessarily represent those of their affiliated organizations, or those of the publisher, the editors and the reviewers. Any product that may be evaluated in this article, or claim that may be made by its manufacturer, is not guaranteed or endorsed by the publisher.

## Footnotes

^1^Following Albarello et al.’s (2019) method, an alternative sequential mediational model was tested reversing the hypothesized sequential mediational path, thus entering identification with the human group, fraternalistic relative deprivation, and attribution of human rights to migrants as mediators of threat’s effect on in-depth exploration of identity in the educational domain. The analysis revealed that the relative total effect of both D_1_ and D_2_ on in-depth exploration of identity in the educational domain was significant (respectively, *B* = −0.59, *SE* = 0.11; *p* < 0.001; *B* = −0.50, *SE* = 0.11; *p* < 0.001). The omnibus test of total effect of D_1_ and D_2_ was significant, *F* (2, 138) = 18.60, *p* < 0.001, *R*^2^ = 0.21. When the mediators were included in the model, the omnibus test of direct effects of D_1_ and D_2_ was still significant, *F* (2, 135) = 14.74, *p* < 0.001, *R*^2^ = 0.16, and the relative direct effects of D_1_ and D_2_ on the dependent variable were still significant, *p*_s_ < 001. Importantly, the sequential indirect effect of threat on in-depth exploration of identity in the educational domain through the three sequential mediators was not significant both for D_1_, *B* = −0.00, *SE* = 0.00, 95% CI [−0.01, 0.01], and D_2_, *B* = −0.00, *SE* = 0.00, 95% CI [−0.00, 0.00].
